# Supplementary figures and images for: Modulatory interactions between the default mode network and task positive networks in resting-state
Source: PeerJ. 2014 May 1;2:e367. doi: 10.7717/peerj.367 (PMC4017816; doi:10.7717/peerj.367)

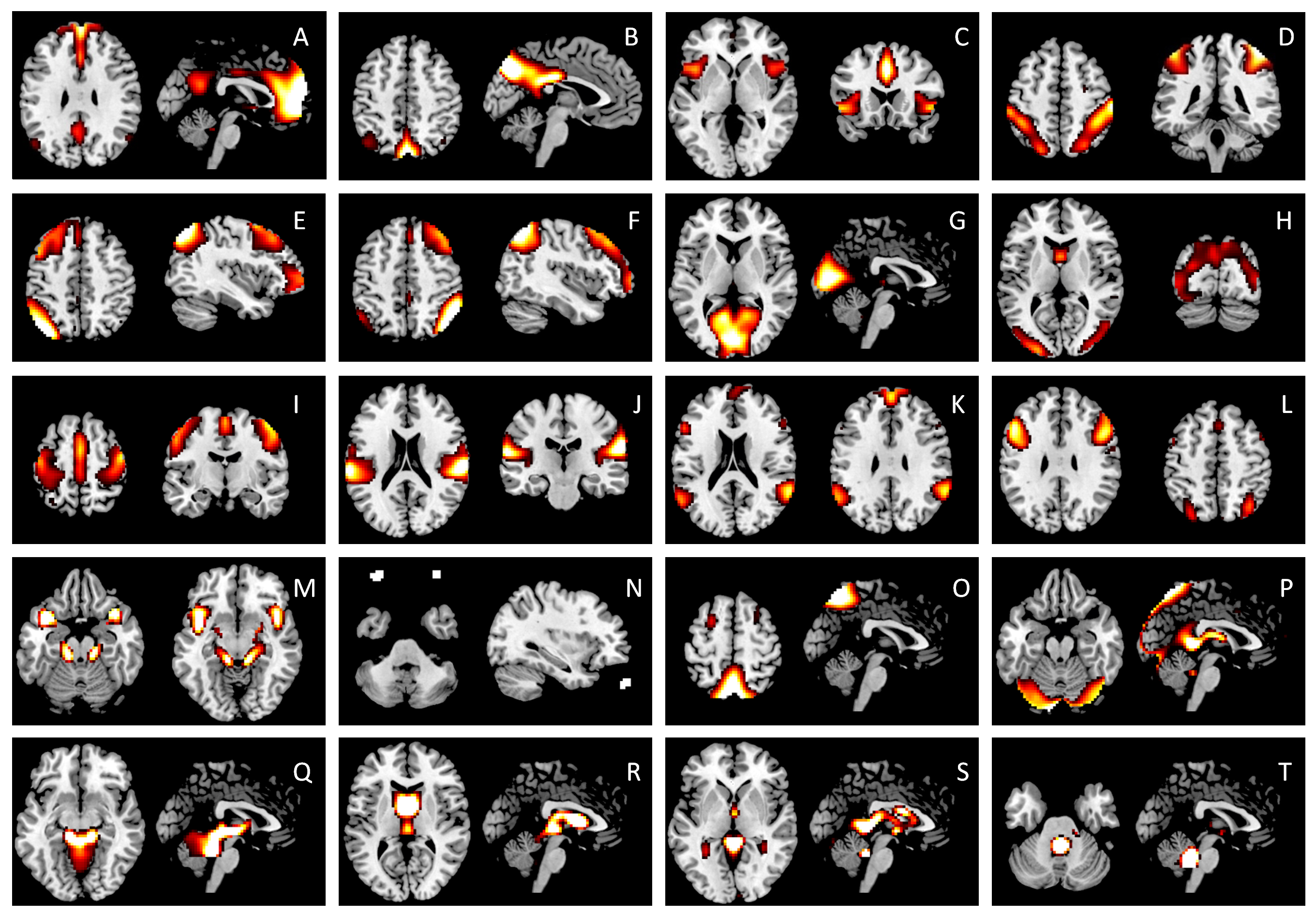

Supplement: Figure S1 — The anterior DMN (A), posterior DMN (B), salience (C), dorsal attention (D), left executive (E), and right executive (F) components were used in the current PPI analysis. ICs G through J are categorized as unimodal networks, including the primary visual (G), higher visual (H), motor (I), and auditory (J) networks. ICs K and L are commonly identified components, however, their functions are not yet been clearly defined. We identified the remaining ICs (M through T) as noises, because their major clusters are located in either large blood vessels, ventricles, or eye balls. The IC maps were z transformed, and thresholded at z > 1.96. [file peerj-02-367-s001.png]
