# Supplementary material for: Modulatory interactions between the default mode network and task positive networks in resting-state
Source: PeerJ. 2014 May 1;2:e367. doi: 10.7717/peerj.367 (PMC4017816; doi:10.7717/peerj.367)
Supplement: Table S1 — Numbers in each cell represent mean beta values and raw p values (in brackets) of corresponding cross subject one sample t-test. Bold font indicates statistically significant after Bonferroni multiple comparison correction of totally 60 correlations. [file peerj-02-367-s002.docx]

**Table S1** Results of the network-wise PPI analysis.

Numbers in each cell represent mean beta values and raw p values (in brackets) of corresponding cross subject one sample t-test. Bold font indicates statistically significant after Bonferroni multiple comparison correction of totally 60 correlations.

|  | Anterior DMN | Posterior DMN | Salience | Dorsal Attention | L. Executive | R. Executive |
| --- | --- | --- | --- | --- | --- | --- |
|  | Anterior DMN |  |  |  |  |  |
| Anterior DMN |  |  |  |  |  |  |
|  |  |  |  |  |  |  |
| Posterior DMN |  |  | 0.026 | 0.017 | -0.016 | **-0.039** |
|  |  |  | (8.36 x 10^-3^) | (0.156) | (0.168) | **(1.48 x 10^-4^)** |
| Salience |  |  |  | 0.008 | 0.032 | **0.060** |
|  |  |  |  | (0.404) | (0.013) | **(1.14 x 10^-5^)** |
| Dorsal Attention |  |  |  |  | -0.013 | 0.006 |
|  |  |  |  |  | (0.216) | (0.560) |
| L. Executive |  |  |  |  |  | -0.009 |
|  |  |  |  |  |  | (0.369) |
|  |  | Posterior DMN |  |  |  |  |
| Anterior DMN |  |  | 0.026 | 0.003 | 0.009 | -0.004 |
|  |  |  | (0.035) | (0.823) | (0.399) | (0.718) |
| Posterior DMN |  |  |  |  |  |  |
|  |  |  |  |  |  |  |
| Salience |  |  |  | 0.022 | 0.001 | 0.012 |
|  |  |  |  | (0.114) | (0.919) | (0.345) |
| Dorsal Attention |  |  |  |  | -0.001 | 0.022 |
|  |  |  |  |  | (0.936) | (0.077) |
| L. Executive |  |  |  |  |  | 0.015 |
|  |  |  |  |  |  | (0.157) |
|  |  |  | Salience |  |  |  |
| Anterior DMN |  | 0.012 |  | 0.021 | 0.009 | **0.054** |
|  |  | (0.271) |  | (0.053) | (0.479) | **(1.25 x 10^-4^)** |
| Posterior DMN |  |  |  | 0.023 | 0.012 | 0.037 |
|  |  |  |  | (0.059) | (0.334) | (2.71 x 10^-3^) |
| Salience |  |  |  |  |  |  |
|  |  |  |  |  |  |  |
| Dorsal Attention |  |  |  |  | 0.003 | -0.009 |
|  |  |  |  |  | (0.791) | (0.463) |
| L. Executive |  |  |  |  |  | 0.025 |
|  |  |  |  |  |  | (0.044) |
|  |  |  |  | Dorsal Attention |  |  |
| Anterior DMN |  | -0.023 | 0.024 |  | -0.024 | -0.014 |
|  |  | (0.109) | (0.024) |  | (0.026) | (0.196) |
| Posterior DMN |  |  | 0.042 |  | -0.013 | 0.022 |
|  |  |  | (1.69 x 10^-3^) |  | (0.323) | (0.060) |
| Salience |  |  |  |  | -0.006 | -0.006 |
|  |  |  |  |  | (0.605) | (0.618) |
| Dorsal Attention |  |  |  |  |  |  |
|  |  |  |  |  |  |  |
| L. Executive |  |  |  |  |  | 0.030 |
|  |  |  |  |  |  | (6.39 x 10**^-3^)** |
|  |  |  |  |  | L. Executive |  |
| Anterior DMN |  | 0.001 | **0.048** | 0.022 |  | -0.009 |
|  |  | (0.966) | **(4.98 x 10^-4^)** | (0.024) |  | (0.374) |
| Posterior DMN |  |  | 0.011 | 0.006 |  | 0.006 |
|  |  |  | (0.331) | (0.593) |  | (0.579) |
| Salience |  |  |  | -0.002 |  | **0.046** |
|  |  |  |  | (0.882) |  | **(1.66 x 10^-4^)** |
| Dorsal Attention |  |  |  |  |  | 0.032 |
|  |  |  |  |  |  | (9.76 x 10^-3^) |
| L. Executive |  |  |  |  |  |  |
|  |  |  |  |  |  |  |
|  |  |  |  |  |  | R. Executive |
| Anterior DMN |  | -0.030 | **0.109** | 0.035 | -0.026 |  |
|  |  | (0.016) | **(1.19 x 10^-11^)** | (4.54 x 10^-3^) | (0.018) |  |
| Posterior DMN |  |  | **0.045** | 0.036 | -0.019 |  |
|  |  |  | **(3.17 x 10^-4^)** | (9.51 x 10^-3^) | (0.084) |  |
| Salience |  |  |  | -0.029 | **0.053** |  |
|  |  |  |  | (0.014) | **(2.06 x 10^-4^)** |  |
| Dorsal Attention |  |  |  |  | **0.058** |  |
|  |  |  |  |  | **(5.91 x 10^-5^)** |  |
| L. Executive |  |  |  |  |  |  |
|  |  |  |  |  |  |  |

L., left.

R., right.
