# Supplementary material for: Modulatory interactions between the default mode network and task positive networks in resting-state
Source: PeerJ. 2014 May 1;2:e367. doi: 10.7717/peerj.367 (PMC4017816; doi:10.7717/peerj.367)
Supplement: Table S2 — Negative t values indicate negative PPI effects. Clusters were thresholded at p < 0.001, and cluster-level false discovery rate (FDR) correction was applied at p < 0.0033, which had taken into account of totally 15 voxel-wise analyses. [file peerj-02-367-s003.docx]

**Table S2** List of regions that showed significant PPI effects in voxel-wise analyses.

Negative t values indicate negative PPI effects. Clusters were thresholded at p < 0.001, and cluster-level false discovery rate (FDR) correction was applied at p < 0.0033, which had taken into account of totally 15 voxel-wise analyses.

| Label | cluster p (FDR) | k | MNI coordinates | | | Peak T |
| --- | --- | --- | --- | --- | --- | --- |
|  |  |  | x | y | z |  |
| Anterior DMN and Salience Network | | |  |  |  |  |
| L. Superior Frontal Gyrus, BA10 | < 0.001 | 2687 | -24 | 53 | 4 | 8.06 |
| R. Precuneus, BA7 | < 0.001 | 667 | 12 | -73 | 61 | 7.13 |
| R. Middle Frontal Gyrus, BA9 | < 0.001 | 898 | 39 | 26 | 28 | 6.37 |
| L. Inferior Parietal Lobule, BA40 | < 0.001 | 1046 | -57 | -43 | 49 | 6.17 |
| L. Middle Temporal Gyrus, BA37 | < 0.001 | 341 | -57 | -52 | -8 | 5.96 |
| L. Posterior Cingulate, BA29 | 0.001 | 77 | -6 | -52 | 13 | 4.47 |
| R. Cingulate Gyrus, BA24 | < 0.001 | 393 | 9 | 11 | 40 | -7.25 |
| R. Lentiform Nucleus, Putamen | < 0.001 | 283 | 21 | 8 | -2 | -6.18 |
| L. Lentiform Nucleus, Putamen | < 0.001 | 247 | -27 | 8 | 10 | -6.16 |
| R. Insula, BA13 | 0.001 | 84 | 54 | -31 | 25 | -5.00 |
| Anterior DMN and Dorsal Attention Network | | |  |  |  |  |
| L. Middle Frontal Gyrus, BA47 | < 0.001 | 529 | -36 | 44 | -11 | 5.93 |
| L. Inferior Parietal Lobule, BA40 | 0.001 | 95 | -45 | -61 | 52 | 5.06 |
| R. Supramarginal Gyrus, BA40 | < 0.001 | 123 | 54 | -40 | 37 | 4.60 |
| R. Superior Frontal Gyrus, BA9 | < 0.001 | 160 | 45 | 41 | 22 | 4.56 |
| Anterior DMN and L. Executive Network | | |  |  |  |  |
| R. Inferior Parietal Lobule, BA40 | 0.003 | 91 | 42 | -37 | 52 | -5.39 |
| Anterior DMN and R. Executive Network | | |  |  |  |  |
| L. Insula, BA13 | < 0.001 | 471 | -45 | 14 | -5 | 6.11 |
| R. Insula, BA13 | < 0.001 | 507 | 36 | 14 | 13 | 5.81 |
| R. Cingulate Gyrus, BA24 | < 0.001 | 227 | 3 | 20 | 28 | 4.77 |
| R. Inferior Parietal Lobule, BA40 | 0.001 | 83 | 66 | -25 | 28 | 4.69 |
| R. Middle Frontal Gyrus, BA10 | < 0.001 | 124 | 36 | 47 | 16 | 4.62 |
| R. Middle Frontal Gyrus, BA6 | 0.001 | 127 | 39 | 17 | 43 | -4.62 |
| Posterior DMN and Salience Network | | |  |  |  |  |
| L. Cingulate Gyrus, BA32 | < 0.001 | 588 | -6 | 41 | 22 | 6.32 |
| L. Cingulate Gyrus, BA31 | < 0.001 | 222 | 0 | -28 | 40 | 5.13 |
| L. Inferior Parietal Lobule, BA40 | 0.001 | 104 | -54 | -52 | 46 | 4.90 |
| Posterior DMN and Dorsal Attention Network | | |  |  |  |  |
| R. Middle Occipital Gyrus, BA19 | < 0.001 | 134 | 36 | -88 | 16 | 6.00 |
| L. Inferior Frontal Gyrus, BA44 | 0.001 | 96 | -54 | 17 | 10 | 5.22 |
| L. Middle Frontal Gyrus, BA47 | < 0.001 | 188 | -48 | 38 | -8 | 5.10 |
| R. Cerebellum, Posterior Lobe | 0.001 | 96 | 27 | -73 | -32 | 4.71 |
| L. Supramarginal Gyrus, BA40 | 0.001 | 106 | -57 | -52 | 40 | 4.50 |
| Posterior DMN and L. Executive Network | | |  |  |  |  |
| n.s. |  |  |  |  |  |  |
| Posterior DMN and R. Executive Network | | |  |  |  |  |
| n.s. |  |  |  |  |  |  |
| Posterior and Anterior DMNs | | |  |  |  |  |
| L. Superior Frontal Gyrus, BA6 | < 0.001 | 238 | -9 | 44 | 49 | -6.59 |
| L. Middle Occipital Gyrus, BA19 | < 0.001 | 165 | -39 | -82 | 19 | -5.31 |
| R. Precuneus, BA7 | 0.001 | 98 | 24 | -70 | 52 | -4.50 |
| Salience Network and Dorsal Attention Network | | |  |  |  |  |
| L. Medial Frontal Gyrus, BA6 | < 0.001 | 433 | 0 | -7 | 58 | 7.45 |
| R. Thalamus | < 0.001 | 254 | 15 | -10 | 16 | 6.01 |
| L. Claustrum | < 0.001 | 188 | -27 | 8 | 13 | 5.34 |
| R. Postcentral Gyrus, BA2 | < 0.001 | 170 | 66 | -16 | 25 | 5.00 |
| L. Inferior Frontal Gyrus, BA9 | < 0.001 | 147 | -42 | 8 | 25 | -4.73 |
| Salience Network and L. Executive Network | | |  |  |  |  |
| L. Medial Frontal Gyrus, BA8 | < 0.001 | 529 | -9 | 41 | 31 | 5.21 |
| L. Superior Temporal Gyrus, BA39 | < 0.001 | 140 | -51 | -55 | 31 | 5.00 |
| L. Middle Frontal Gyrus, BA6 | 0.002 | 84 | -48 | 14 | 46 | 4.58 |
| Salience Network and R. Executive Network | | |  |  |  |  |
| R. Superior Frontal Gyrus, BA8 | < 0.001 | 1219 | 3 | 44 | 43 | 5.46 |
| R. Inferior Frontal Gyrus, BA47 | < 0.001 | 138 | 48 | 35 | -8 | 5.46 |
| R. Superior Temporal Gyrus, BA39 | < 0.001 | 197 | 54 | -52 | 34 | 5.18 |
| R. Precentral Gyrus, BA9 | < 0.001 | 137 | 36 | 17 | 31 | 5.05 |
| Dorsal Attention Network and L. Executive Network | | |  |  |  |  |
| L. Inferior Parietal Lobule, BA40 | < 0.001 | 186 | -48 | -55 | 55 | 5.46 |
| L. Middle Frontal Gyrus, BA6 | < 0.001 | 112 | -45 | 14 | 46 | 4.95 |
| Dorsal Attention Network and R. Executive Network | | |  |  |  |  |
| R. Precuneus, BA39 | < 0.001 | 160 | 45 | -70 | 40 | 4.43 |
| L. Executive Network and R. Executive Network | | |  |  |  |  |
| L. Precuneus, BA7 | < 0.001 | 410 | -30 | -70 | 58 | 5.20 |
| R. Precuneus, BA7 | 0.001 | 99 | 27 | -73 | 58 | 4.67 |

MNI, Montreal neurology institute.

L., left

R., right

n.s., not significant
